# Bumble bees in landscapes with abundant floral resources have lower pathogen loads

**DOI:** 10.1038/s41598-020-78119-2

**Published:** 2020-12-18

**Authors:** Darin J. McNeil, Elyse McCormick, Ashley C. Heimann, Melanie Kammerer, Margaret R. Douglas, Sarah C. Goslee, Christina M. Grozinger, Heather M. Hines

**Affiliations:** 1 Department of Entomology, Insect Biodiversity Center, Center for Pollinator Research, Huck Institutes of the Life Sciences, Pennsylvania State University, University Park, PA 16802 USA; 2 Department of Biology, Pennsylvania State University, University Park, PA 16802 USA; 3 Department of Environmental Studies and Environmental Science, Dickinson College, Carlisle, PA 17013 USA; 4 United States Department of Agriculture-Agricultural Research Service, Pasture Systems and Watershed Management Research Unit, University Park, PA 16802 USA

**Keywords:** Conservation biology, Macroecology, Population dynamics, Biodiversity, Ecological modelling

## Abstract

The pollination services provided by bees are essential for supporting natural and agricultural ecosystems. However, bee population declines have been documented across the world. Many of the factors known to undermine bee health (e.g., poor nutrition) can decrease immunocompetence and, thereby, increase bees’ susceptibility to diseases. Given the myriad of stressors that can exacerbate disease in wild bee populations, assessments of the relative impact of landscape habitat conditions on bee pathogen prevalence are needed to effectively conserve pollinator populations. Herein, we assess how landscape-level conditions, including various metrics of floral/nesting resources, insecticides, weather, and honey bee (*Apis mellifera*) abundance, drive variation in wild bumble bee (*Bombus impatiens*) pathogen loads. Specifically, we screened 890 bumble bee workers from varied habitats in Pennsylvania, USA for three pathogens (deformed wing virus, black queen cell virus, and *Vairimorpha* (= *Nosema*) *bombi*), *Defensin* expression, and body size. Bumble bees collected within low-quality landscapes exhibited the highest pathogen loads, with spring floral resources and nesting habitat availability serving as the main drivers. We also found higher loads of pathogens where honey bee apiaries are more abundant, a positive relationship between *Vairimorpha* loads and rainfall, and differences in pathogens by geographic region. Collectively, our results highlight the need to support high-quality landscapes (i.e., those with abundant floral/nesting resources) to maintain healthy wild bee populations.

## Introduction

The pollination services provided by bees are critical for supporting healthy and diverse natural and agricultural ecosystems^1^. Continuing declines documented in populations of wild and managed bees across the world thus pose significant threats to the stability of these systems^2,3^. Declines in bee populations have been attributed to several factors^4^. Extensive habitat loss and degradation results in a dearth of floral resources and nest sites which has contributed to loss of wild bee abundance and diversity^5,6^. Bee losses, especially those of honey bees (*Apis mellifera*) and bumble bees (*Bombus* spp.), have more recently been ascribed to rising levels of novel bee pathogens^3,7^. For example, the microsporidian *Vairimorpha* (= *Nosema*) *bombi* (hereafter, *Vairimorpha*) has been identified as a major contributor to bumble bee population losses, leading to the extirpation and near extinction of several bumble bee species in North America^8,9^. Several bee viruses also contribute to declining honey bee survival^7^. These honey bee viruses often spill over into native bee populations^10–12^, where they impose negative effects on wild bee health^7,13^. In the US, the toxic load to bees of insecticides applied in agricultural landscapes has increased significantly over the last few decades^14^. Exposure to insecticides, particularly neonicotinoids, can lead to a variety of negative effects on native bees and managed honey bees alike, including compromised learning and foraging capabilities and reduced reproductive output^2,15–17^. More recently, climate change has been added to the list of major threats to global bee populations, as large-scale geographic analyses identified climate shifts as a contributing factor to local bumble bee extirpation^18^. Given the myriad of threats to pollinator populations, it can be difficult to tease apart the relative importance of each of these factors to bee health.

Determining the leading threats to bee populations is further complicated by interactions among these stressors in the landscape, specifically many of the factors known to undermine bee health (such as poor nutrition or exposure to pesticides) can increase susceptibility to disease^19^. Bees are more likely to be nutritionally deprived in landscapes with fewer and less diverse flowering plants, and nutritional deprivation can reduce immunocompetence and increase pathogen and parasite load^19,20^. For example, honey bees fed diets lacking pollen had higher loads of viruses^21^ and diets with low pollen species diversity increased honey bee mortality when bees were infected with *Vairimorpha*^22^. Caged bumble bees (*B. terrestris*) exhibited increased mortality from viruses only if starved^23^. Nutritional deprivation is likely to act through altering immune function, as immune genes are more highly expressed when forage is abundant^24^.

Pesticide exposure can also reduce bee immunocompetence, resulting in increased susceptibility to pathogens and parasites^25^. Honey bees have higher viral loads after exposure to neonicotinoids^26^, K_ATP_ channel agonists^27^, and organosilicones^28^, with suppression of immune genes implicated in this response in neonicotinoid-fed bees^26^. Acaricides have been found to increase mortality in virus-infected bees^25^ and exposure to a neonicotinoid and fungicides increased loads of *Vairimorpha* in honey bees^29,30^. Similarly, a study in bumble bees using data compiled from across the United States found that *Vairimorpha* prevalence in declining bumble bee species is best predicted by usage of the insecticide clothianidin^31^.

The incidence and loads of a particular pathogen or parasite in bee populations is also likely influenced by bee population density and the composition of bee communities. Viruses, ectoparasites (such as mites) and endoparasites (including *Vairimorpha* spp.) can be transmitted through interactions from parent to offspring, within colonies of social bees, or within and between bee species when bees co-forage on flowers^7,32^. Landscapes in which floral resources are limited can have increased transmission risk of pathogens and parasites through more extensive sharing of limited resources^33^, and viral prevalence in honey bees and bumble bees has been shown to increase with colony density^34^. Similarly, as most viruses can be shared among individual bees and even different species^10,35^, incidence of viruses and parasites in wild bumble bees has been found to be heavily influenced by the presence of honey bees, which often harbor high pathogen loads^12,36^.

Given all these interacting factors, disease prevalence and virulence in a population of bees can be challenging to model or predict in wild bee populations^37^. Quantifying how different stressors in the landscape impact bee disease pathogen prevalence in the wild would mark an important step in our understanding of pollinator disease ecology. Much of the previous work laying the foundation for our understanding of the factors influencing bee health has been lab-based. Some small-scale studies (e.g., Refs.^38,39^) have helped to understand how pathogen patterns manifest in the field, however, understanding how disease patterns arise in nature remains largely theoretical. Understanding of which factors are most likely to contribute to pathogen loads in the wild may be best gained through examining trends occurring across larger, more diverse landscapes (cf., Refs.^18,32^). Herein we evaluate wild bee pathogen loads to assess the relative role of diverse landscape characteristics on infectious disease prevalence, and more generally bee health, in wild bumble bee populations. In particular, we explore how three pathogens (deformed wing virus (DWV), black queen cell virus (BQCV), and *Vairimorpha* (= *Nosema*) *bombi*), as well as expression of an immune gene that upregulated in response to a range of pathogens (*Defensin*^40^), vary in wild bumble bees (*B. impatiens*) over two years and across varied landscapes in Pennsylvania, USA. We examined the extent to which several potential landscape-scale stressors—floral abundance, nesting habitat quality, insecticide loading, climatic factors, and interactions with managed honey bees—drive variation in pathogen loads in bumble bees. Not only do such analyses allow us to examine the environmental factors contributing to pathogen loads, but, given that bee disease is so influenced by environmental stressors, loads of bee pathogens in populations can be an indicator of which environmental factors may be most detrimental to overall bee health.

## Results

### Pathogen incidence

**Figure 1:**
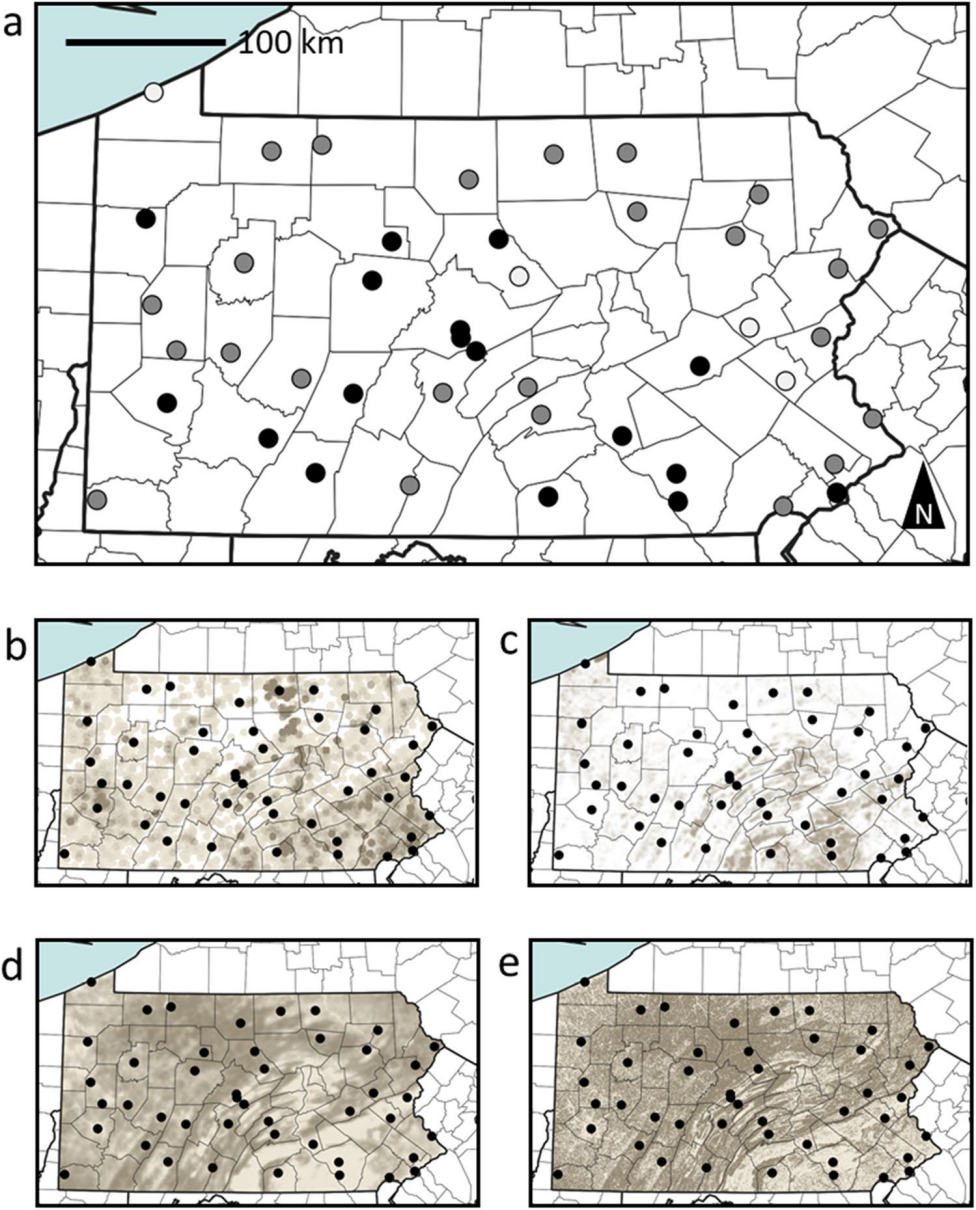
Map depicting locations (circles) where we sampled *Bombus impatiens* workers across 38 counties in Pennsylvania. Hollow circles indicate locations where sites were sampled in 2018 only, shaded circles indicate sites sampled in 2019 only and black circles indicate locations sampled in both years (**A**). Also shown are spatial patterns of several important habitat covariates: honey bee colony density (**B**), agricultural insecticide loading (**C**), spring floral resources (**D**), and forest cover (**E**). For maps (**B**–**E**), darker shades indicate higher values for each variable.

**Figure 2:**
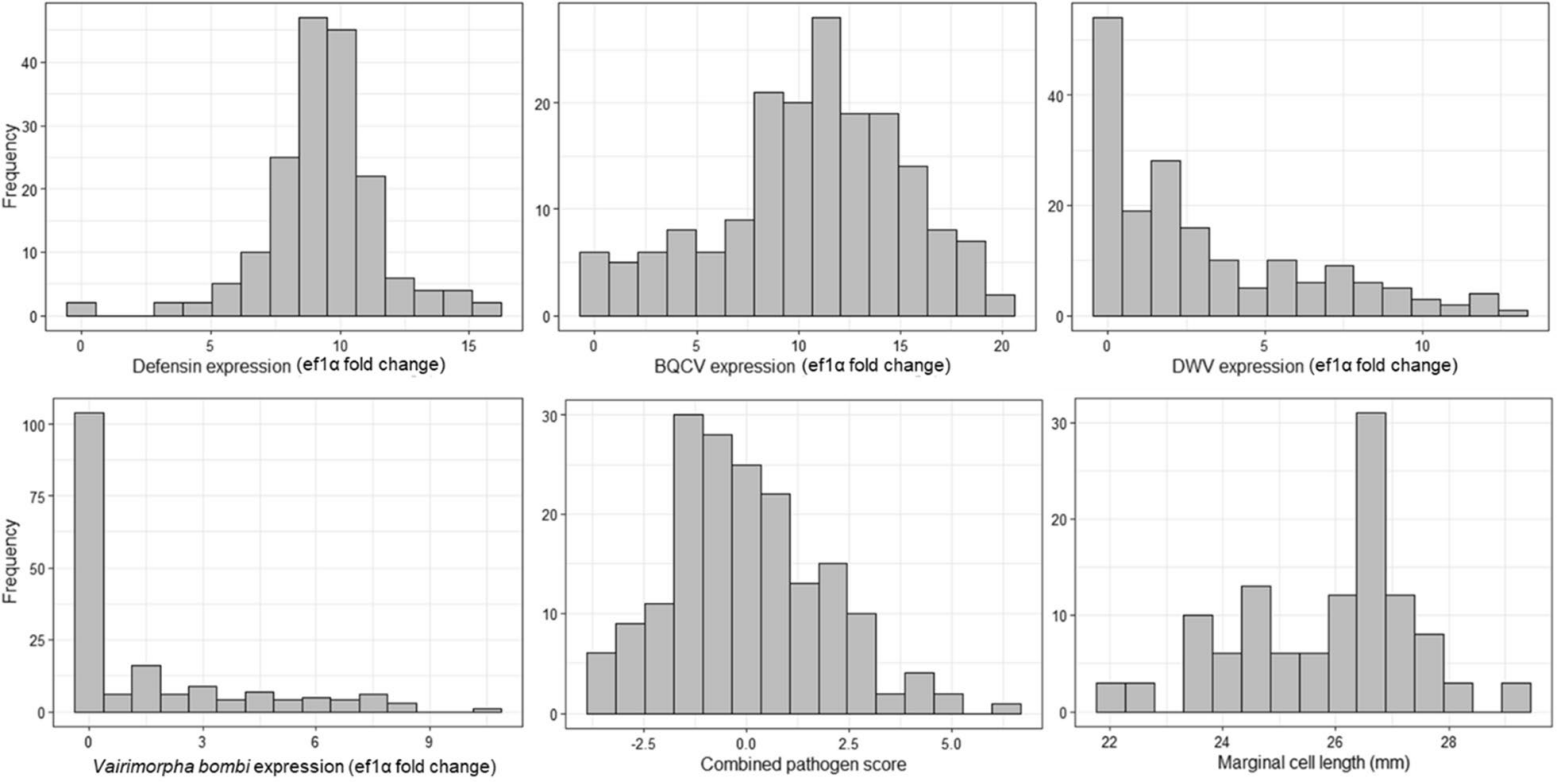
Histograms of the six response variables evaluated in *Bombus impatiens* workers across 38 counties in Pennsylvania: *Defensin* (top left), black queen cell virus (BQCV) (top center), deformed wing virus (DWV) (top right), *Vairimorpha* (bottom left), combined pathogens (bottom center; scaled to zero) and marginal cell length (bottom right). Shown on the X-axes are normalized expression values for DWV, BQCV, *Vairimorpha* and defensin, normalized to the amount of EF-1α in each sample.

For pathogen analysis, we collected workers of *B. impatiens*, as it is the most abundant species across our Pennsylvania study region. We collected 890 workers from sites spanning a diversity of habitats across Pennsylvania, USA (Fig. 1A), for 2–3 weeks during the peak of bumble bee abundance (late June–mid July), and across 2 years, including 21 sites in 2018 (n = 310 bees) and 41 sites in 2019 (n = 580 bees). Screens of loads of pathogens among pooled bees (n = 5 bees/pool) from each site (typically 3 pools/site), performed via quantitative PCR, revealed that most bumble bee samples (97%) contained BQCV and that BQCV exhibited a broad range of pathogen load values. DWV was much less common, with several samples (29%) lacking notable DWV detection (Fig. 2), and most of those samples that did test positive for DWV exhibiting low loads. Honey bee samples from the same sites (unpublished data) had much higher loads of DWV, suggesting that low loads were not due to issues with our detection protocol, but rather that *B. impatiens* tends not to harbor high loads of this pathogen. *Vairimorpha* detections were sporadic, detected in about 40% of samples, but usually at low loads. All bumble bee samples exhibited some degree of expression of the immune gene, *Defensin* (Fig. 2).

### Landscape correlates with pathogen loads

**Figure 3:**
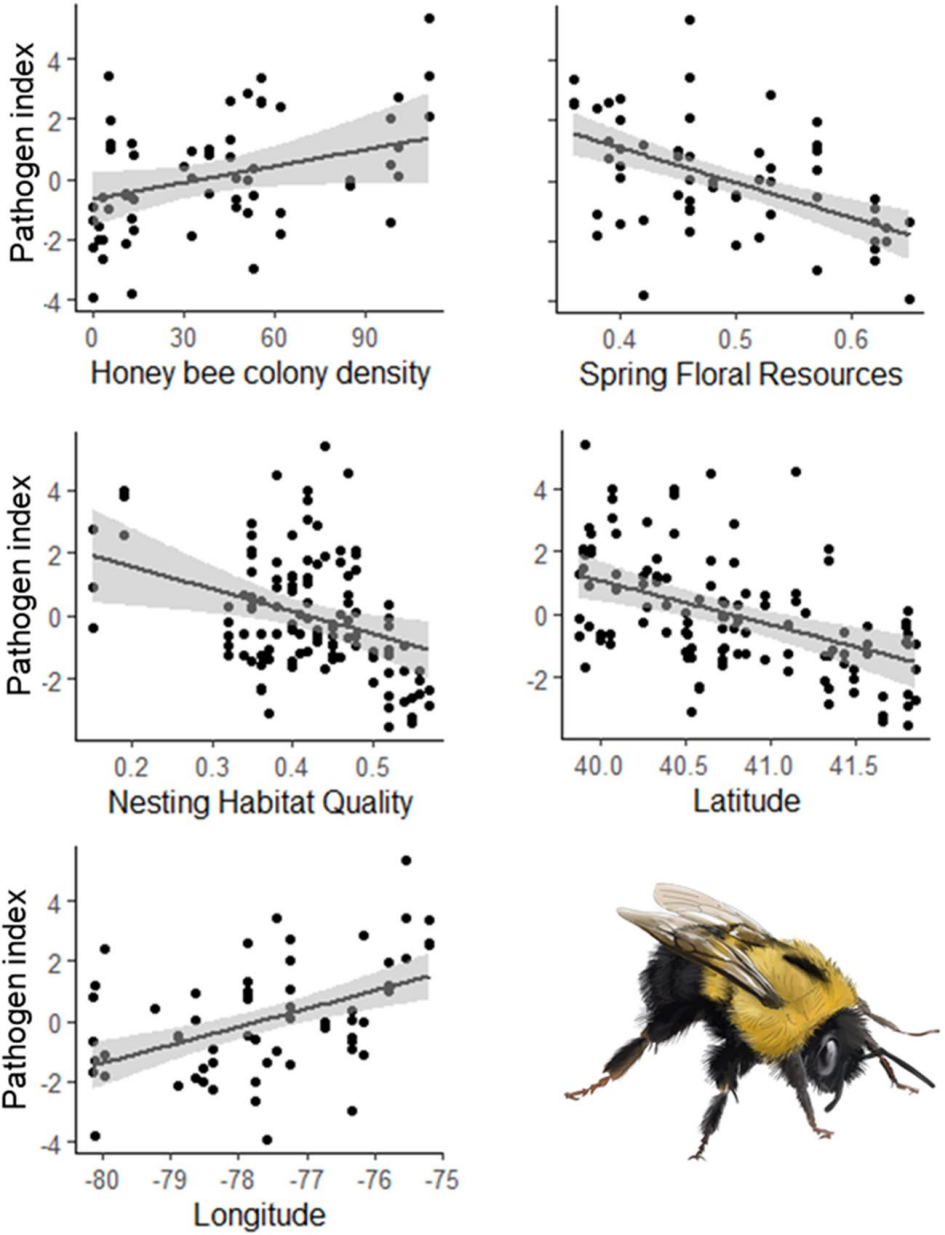
Functional relationships for mixed-effects regression analyses of bumble bee (*Bombus impatiens*) pathogen loads across Pennsylvania landscapes (2018–19). Shown are relationships for our ‘combined’ pathogen index (scaled) which included *Vairimorpha*, black queen cell virus, and deformed wing virus infection. Only patterns with statistical support based on Akaike’s Information Criterion are shown. Points on each graph indicate data points while black lines indicate model estimates and gray shaded regions are 95% confidence intervals. Pathogen loads were regressed against honey bee colony density (# colonies within 5 km; 2018; top left), spring floral resources (2018; top right), nesting habitat quality (2019; center left), latitude (2019; center right) and longitude (2018; bottom left).

Pathogen loads inferred for DWV, BQCV, *Vairimorpha*, and a combined pathogen index were modeled against several landscape predictor variables in two separate modeling analyses (tiers) using ranked candidate sets of linear mixed-effects models (see “Statistical analyses” section, below). Our first model tier (Tier 1) examined associations between pathogen loads and key landscape indices: floral resource quality in spring and summer, nesting resource quality, insecticide loading, and honey bee abundance, with each year modeled separately. Tier 1 models revealed higher loads of BQCV at sites with lower quality spring floral resources (both years), lower quality nesting resources (2018), and higher honey bee colony density (2019; Supplementary Table S2). These models suggested higher DWV loads at sites with lower quality summer floral resources (2018), lower quality nesting resources (2019) and intermediate insecticide exposure (2019; Supplementary Table S4). *Vairimorpha* infection had no strong covariate relationships, as a null model was supported in Tier 1 models (Supplementary Table S3). Our Tier 1 combined pathogen index indicated higher *B. impatiens* pathogen loads with lower quality spring floral resources (2018), lower quality nesting resources (2019), and higher honey bee colony densities (2018; Fig. 3; Supplementary Table S5). These results resemble the BQCV results, which is not surprising given that BQCV had the highest infection rates among the three pathogens. Tier 1 *Defensin* expression in both years indicated support for a null model, suggesting no strong covariate relationships with any predictor (Table 1; Supplementary Table S1), and our analyses revealed no relationship between Tier 1 covariates and marginal cell size (Table 1; Supplementary Table S6), suggesting that *B. impatiens* body size was not significantly influenced by these landscape variables.

**Table 1:** Habitat variables associated with metrics of bumble bee (*Bombus impatiens*) health across varied landscapes in Pennsylvania 2018–19.

*Bombus* health metric	Habitat variable
**Tier 1 Habitat Analyses**	
*Defensin* expression	–
BQCV infection	Spring floral resources (−) Nesting resources (−) Honey bee colony density (+)
*Vairimorpha* infection	–
DWV infection	Spring floral resources (+) Summer floral resources (−) Nesting resources (−) Insecticide loading (−)
Combined pathogen score	Spring floral resources (−) Nesting resources (−) Honey bee colony density (+)
Marginal cell length	–
**Tier 2 Habitat Analyses**	
Defensin expression	Grassland/pasture cover (+)
BQCV infection	Longitude (+) Latitude (−) Forest cover (−) Natural cover (−) Developed cover (+)
*Vairimorpha* infection	Spring precipitation (+)
DWV infection	Latitude (−) Arable cover (−)
Combined pathogen score	Longitude (+) Latitude (−)
Marginal cell length	–

Variables shown below are those with statistical support from our mixed-effects regression analyses (Appendices 1–6) based on Akaike’s Information Criterion adjusted for small sample size and *β* coefficient 95% confidence intervals (those overlapping zero considered to be weak relationships) in either year.

Our second model tier considered the same covariates as model Tier 1, and also considered additional variables that might be useful predictors of bumble bee health: latitude, longitude, elevation, weather variables, catch-per-effort, local *Bombus* spp. diversity (H′; measured from the first 20 workers collected per site) and land cover variables (i.e., NLCD percent cover). These models revealed positive relationships between BQCV loads and both longitude (2018) and developed land cover (2018), indicating that more eastern- and developed sites hosted the most BQCV-infected bumble bees (Supplementary Table S2). Likewise, we found relationships between BQCV loads and latitude (negative; 2018), longitude (positive; 2018), forest cover (negative; 2018), natural cover (negative; 2018), developed cover (quadratic; 2018), shrubland cover (negative; 2019), honey bee colony density (positive; 2019), spring growing degree days (quadratic; 2019), summer floral resources (negative; 2018), and spring floral resources (negative; both years; Supplementary Table S2). DWV was most prevalent in sites with low arable cover (2019), intermediate insecticide loading (2019), sparse summer floral resources (2018), high longitudes (i.e., eastern; 2018) and low latitudes (2019; i.e., southerly sites; Supplementary Table S4). Further, our analysis demonstrated that sites with more spring rain hosted bees with greater *Vairimorpha* loads (2019; Supplementary Table S3). Tier 2 model sets for *Defensin* expression indicated a positive relationship with percent grassland/pasture cover in 2018 only (Table 1; Supplementary Table S1). Our Tier 2 combined pathogen models indicated that latitude (negative; 2019), longitude (positive; 2018) and spring floral resources (negative; 2018) were the best predictors of overall pathogen loads in *B. impatiens* across Pennsylvania (Fig. 3; Supplementary Table S5). Finally, as with Tier 1 analyses, we found no relationship between Tier 2 covariates and marginal cell size (Table 1; Supplementary Table S6) suggesting that *B. impatiens* body size varied independently with respect to covariates in either model tier. Similarly, no significant relationships were found between pathogen loads and bee species diversity or bee abundance per unit sampling time.

**Figure 4:**
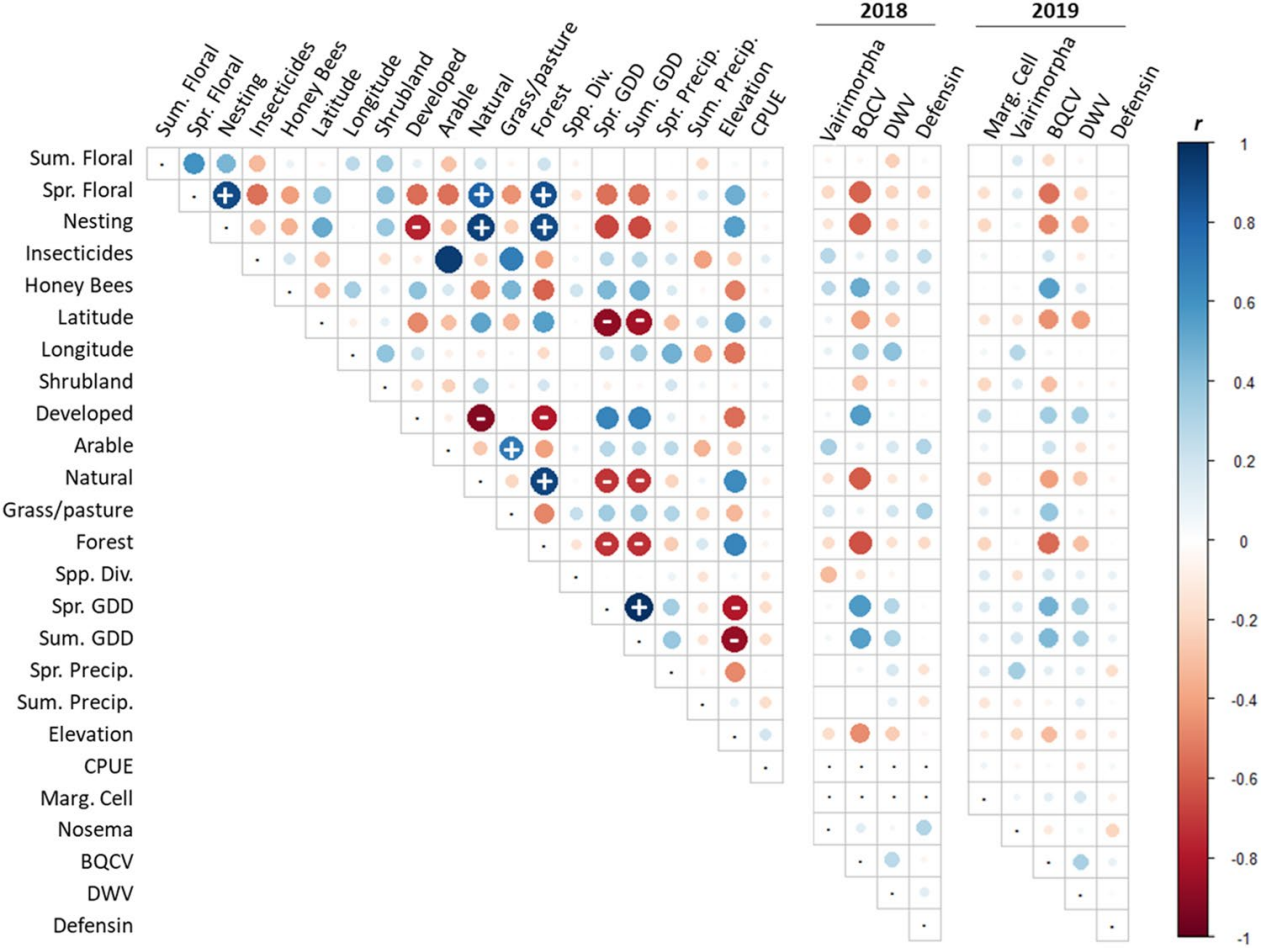
Correlation matrix for all pairwise independent- (2019 shown) and response variables in 2018 (center) and 2019 (right) for *Bombus impatiens* workers from 38 counties in Pennsylvania. Values within each cell represent Pearson’s Correlation Coefficient (*r*). Circle size indicates the magnitude of *r* with values ≥ 0.70 denoted with a sign overlaying each circle. Habitat variables shown include summer floral (“Sum. Floral”), spring floral (“Spr. Floral”), nesting resources (“Nesting”), insecticide loading (“Insecticides”), honey bee (*Apis mellifera*) colony density (“Honey Bees”), latitude, longitude, percent shrubland (within 500 m; “Shrubland”), percent urban development (“Developed”), percent arable (“Arable”), percent natural (“Natural”), percent grassland/pasture (“Grass/pasture”), percent forest (“Forest”), and elevation. Additionally shown are weather variables: spring growing degree days (“Spr. GDD”), summer growing degree days (“Sum. GDD”), amount of spring precipitation (“Spr. Precip.”), and amount of summer precipitation (“Sum. Precip.”). Finally, we also included *Bombus* spp. standardized Shannon diversity (H′; “Spp. Div.”) and number of *B. impatiens* captures per hour/observer (“CPUE”). Response variables included forewing marginal cell size (“Marg. Cell”), *Vairimorpha* infection, black queen cell virus infection (“BQCV”), deformed wing virus infection (“DWV”), and *Defensin* expression.

Although the variables that were most significant varied between years by pathogen, most trends found in both Tier 1 and Tier 2 variables were consistent in directionality between years, indicating that results were repeatable across years (Fig. 4). These models sought to find the variables that explained the data the most, allowing up to 2 variables to be combined within a model, without including correlated variables in a given combinatorial model. Several of our variables, however, were correlated and may alternatively explain patterns. For example, percent ‘natural’ cover was positively correlated (|*r|*> 0.70) with the spring floral resource and nesting indices, as well as percent forest cover (Fig. 4). These two variables were also both negatively correlated with percent ‘arable’ land cover. Spring GDD and summer GDD were positively correlated and both were negatively correlated with percent forest cover, percent natural cover, latitude, and elevation (Fig. 4).

## Discussion

Our study is among the first to evaluate how different aspects of landscape quality correlate with the distribution and loads of key pathogens and parasites in wild bees. We used generalizable landscape indices of key landscape characteristics known to influence bee health (e.g., forage resource quality, nesting resource quality, and insecticide toxic loads) in our analyses, which allowed us to assess pathogen load patterns over diverse and geographically distributed landscapes. Our results demonstrate that bees sampled from lower quality landscapes have higher loads of pathogens and parasites, with spring floral resources, nesting habitat availability, and honey bee colony density driving the strongest patterns. While clearly these patterns need to be verified across a larger spatio-temporal scale and with more bee species, our results suggest it may be possible to predict potential risks from pathogens and parasites based on these landscape indices. These indices and models can thus help inform decisions as to where habitat restoration and conservation practices should be applied.

Inadequate forage (i.e., floral dearth) has been cited, along with emerging infectious disease, as among the most important threats to global bee populations^4^. While these two stressors clearly affect bees independently^32,41^, our study provides support for the hypothesis that nutritional stress (via lack of floral resources) and disease infection may also interact to impact wild bee populations^19,42^. This is consistent with laboratory-based studies that suggest nutritionally-stressed bees exhibit compromised immunocompetence^38^ and greater pathogen loads^43^, and analogous results have been reported in vertebrate systems^44,45^. Additionally, wild bees may benefit from having a choice of floral secondary plant compounds that may be constrained in conditions of floral dearth, as some such compounds are known to reduce pathogen loads^32^. Although a negative relationship between floral availability and disease infection seems intuitive, alternative hypotheses predict the opposite pattern whereby abundant floral resources facilitate more interaction between managed- and wild bee populations^4,46^. Although floral resources were strongly correlated with pathogen loads, we were surprised that features like urban/agricultural development did not predict pathogen loads (Table 1). One potential explanation for this pattern was that such habitats host adequate floral resources to support healthy wild bee populations^47^. Our results indicate that spring resources, as opposed to summer resources, were more strongly correlated with pathogen loads. The spring is a vulnerable period for bumble bees, as resources can be patchy and phenologically mismatched, while queen bees emerge partially depleted of resources from diapause and must find sufficient resources to successfully start their colonies^48^. More attention should be drawn to the abundance of spring resources for supporting bumble bees and other wild bees.

Honey bees, which are not native to North America, are well-known disease vectors with the potential to impact wild bees through the introduction of novel pathogens or promotion of those already present within populations^4,35^. In further support of this, our analyses reveal a positive correlation between managed honey bee colony density and some bumble bee pathogen loads (Table 1). Honey bees host high pathogen loads as compared to ‘background’ wild bee populations, especially for DWV and BQCV (but not *Vairimorpha bombi*^12,36^). Pathogen loads present within managed honey bee colonies can be transmitted to wild bees through honey bee foraging activities which usually extend several kilometers around each colony^49^. While our analysis suggested that higher honey bee density was associated with higher bumble bee pathogen loads, patterns varied by year and disease. Although directionality of honey bee density effect was similar across pathogens and years, honey bee impacts were only significant in 2018 and only for BQCV and our combined pathogen index, but not DWV, *Vairimorpha*, or our non-pathogen metrics (*Defensin* and marginal cell length). Moreover, our tier 2 models indicated that variables like latitude and longitude were better explanators of pathogen loads than was honey bee colony density. It is important to note that the presence of viruses commonly found in honey bees within wild bees is not necessarily indicative of pathogenicity, though it is possible that these viruses can become more problematic if wild bees are stressed or immunocompromised due to other factors^13,50^.

Pesticides pose a significant threat to wild bee populations^4,51^ and the aggregate hazard of insecticides applied to agricultural landscapes across North America has risen in recent decades as a result of widespread use of neonicotinoid seed treatments in common field crops^14^. However, our analyses revealed few clear relationships between pathogen loads and insecticide loading, with several possible explanations. To observe an impact on bumble bee pathogen loads, pesticide exposure likely needs to be high enough to suppress bees’ immune systems while remaining sublethal^42^. To this end, we may not expect a linear increase in pathogen loads with increasing insecticide loading because highly toxic sites have few bees^42^. Furthermore, Pennsylvania may not have enough variation in insecticide use across our study sites (Fig. 1C) because most of the state is forested^52^ and such natural habitats typically have relatively low insecticide loads^53^. Finally, the insecticide index considered here is based on annual, state-wide insecticide application data for croplands, and it is possible that field- and time-specific insecticide data—or data including non-agricultural insecticide uses—may reveal clearer relationships with pathogen loads. Still, even under the best circumstances, linking pesticides to bee health has proven challenging for observational studies like ours^51^.

In this study, we quantified expression of an immune gene *Defensin*, considering that this may be a direct metric of overall pathogen strain on the bee. *Defensin* is one of four antimicrobial peptides produced in bees as an immune response to pathogen infection from bacteria, protozoa, fungi, and viruses, and thus can serve as an indicator of pathogen load^40^. We, however, found that *Defensin* expression did not correlate well with any of our landscape factors. The use of immune genes for understanding pathogen loads has had mixed results in the literature, as high levels of nutritional resources can boost both immunocompetence and immune gene expression^40^. Immune gene expression in response to pathogens is therefore likely to be complex.

One of the most consistent patterns we observed in our study was that pathogen loads varied geographically across our study area. This finding echoes the spatially heterogeneous disease patterns reported for wild bees by other studies^12,32^. With this in mind, latitude and longitude are likely proxies for variables (or suites of variables) not fully measured in our study. For instance, both BQCV and DWV were least common in northern Pennsylvania. Indeed, northern Pennsylvania supports a suite of landscape characteristics expected to support healthy bee populations; these landscapes tend to support more native habitat, host better floral resources, and fewer managed honey bee colonies (Fig. 1B–E). Indeed, past predictive models of bee abundance suggest northern Pennsylvania as among the highest quality regions in the eastern United States^54^ and our data seem to support this notion, at least from a disease perspective. Ultimately, the geographic variation we observed in bumble bee pathogen loads during our study highlights the importance of accounting for regional variation in assessments of disease risk for wild bees, especially for widely-dispersed species like *B. impatiens*^55^.

Weather patterns can have profound impacts on animal disease dynamics because weather impacts disease agents, vectors, and even host activity^56,57^. For example, Retschnig et al.^58^ observed a negative relationship between ambient temperature and *Vairimorpha* infection rates in honey bee colonies because colder temperatures kept bees from foraging outside the colony and within close proximity to nest-mates. The opposite pattern has been reported for DWV virus titers across a temperature gradient^59^, however, higher temperatures also reduced bee survival rates. Given the complexity of relationships between insect pathogens and environmental conditions, it is not entirely surprising that our models suggested only modest correlations between weather and pathogen loads (Table 1). The clearest pattern we observed was a positive relationship between spring precipitation and infection by *Vairimorpha bombi*. Indeed, several previous studies have indicated that spore viability and infection for other *Vairimorpha* spp. to be positively correlated with rainfall (e.g., Ref.^60^), presumably because rainfall enhances transmission rates^61^ (but see Ref.^62^). Future studies should model more detailed weather parameters than we have to examine their relative impacts.

Although our study marks an important exploration into the macro-ecological patterns of pathogen prevalence in a wild bee population, it is important to keep several important caveats in mind with the interpretation of our results. We observed year-to-year variation in the strength, but not direction, of habitat trends (i.e., Fig. 4). The consistency in general patterns highlights the value of multi-year studies for revealing reliable trends, but the variance suggests that we may have uncovered additional patterns had our study continued beyond two years. Additionally, Pennsylvania is a highly forested state^51^; analyses of data similar to those presented here from other regions with different ecosystem types (e.g., grasslands, intense agriculture, etc.) would help to decouple co-correlated variables and better understand the relative impacts of different habitats. Furthermore, we did not sample pathogens from sites with very few bees (i.e., those where we could not collect ≥ 5 *B. impatiens*), which could have hampered our ability to assess some of the effects of the lowest quality sites on bees. Finally, many variables in our analyses were correlated (|*r|*≥ 0.70) and could not be modeled simultaneously. Because correlated variables were included as separate variables in each tier, competing models often included different members of a set of correlated variables (e.g., Supplementary Table S2), indicating uncertainty as to which variable best predicted disease loads. Future analyses might consider methods that account for correlated variables (e.g., ordination) or are robust to variable correlation (e.g., random forest, etc.).

Collectively, our results highlight the need to support high-quality landscapes (i.e., those with abundant floral/nesting resources) to maintain healthy wild bee populations. These results are particularly timely in light of widespread population declines in many insect groups^63^, especially pollinators like bumble bees^2^. In addition to helping support healthy pathogen-free bees, conservation of low-pathogen habitats identified here is also important from other perspectives; landscape features that support abundant floral and nesting resources also host more abundant and diverse pollinator populations and communities^54,64^, and the critical ecosystem services they provide (Russo et al. 2013). The model results presented here could also be mapped across landscapes and incorporated into conservation/planning tools (e.g., The ‘Beescape’ program; beescape.org). Indeed, spatially explicit conservation tools like Beescape that map biological patterns in space can be instrumental in ensuring conservation success for sensitive species like bees^65,66^. With that in mind, the analytical approaches used here coupled with the habitat indices we incorporated could be applied to assess wide array of conditions and study areas, thus allowing even broader insights to relative value of different landscape features for pollinators.

## Methods

### Site selection

Our sample sites (21 sites in 2018 and 41 sites in 2019) includes 38 of the 67 counties across Pennsylvania (Fig. 1A). These sites were selected to represent evenly the span of the state while also collecting from a wide suite of habitat types and land use patterns. The central Appalachian Mountains run through the center of the state and, therefore, vary in elevation from relatively low (~ 100–200 m) to moderately high (> 500 m^67^). Pennsylvania is heavily dominated by mature forest^68^ with northern hardwood (e.g., *Acer* spp.), Appalachian oak (*Quercus* spp.), and coniferous (e.g., *Tsuga canadensis*) among the most common forest types^69,70^. Although high-elevation regions in Pennsylvania remain largely forested^52^, lower elevations host fertile soils on which row cropping and livestock-based agriculture are important land uses, as is human development^71^. As a result, a range of values for each of nesting habitat, floral resources, and agricultural insecticide loading was available for sampling (Fig. 1).

### Bee sampling

Upon arriving at each site, we conducted an unlimited-length visual encounter survey (VES) for bumble bees, where 1–2 surveyors examined all available flowers as evenly as possible for bumble bees, ignoring bumble bee species identity, until we caught 20 *Bombus* workers. In most sites, bees were captured within 100–200 m of the site centroid. Gathered bumble bees were identified in the field and retained for lab identification if identity was uncertain (rarely necessary). Given the difficulty of identifying *B. perplexus*, *B. vagans*, and *B. sandersoni* workers, these were lumped and treated as a single taxon. This 20-bee survey allowed us to obtain a diversity metric for each site (standardized Shannon Diversity Index (H′)^72^). All bees were captured legally under Pennsylvania special use permit #2019-75.

For pathogen analysis, we sought to collect 15 *B. impatiens* from each site; if our initial sample of 20 *Bombus* included fewer than 15 *B. impatiens* workers, we continued sampling until 15 *B. impatiens* workers were collected. During 2019 sampling, we recorded the start- and end times at each site which allowed us to calculate ‘catch per unit effort’ for *B. impatiens* in 2019 as: (# *B. impatiens* within the first 20 bees sampled/survey duration)/the number of surveyors. A few sites where 15 *B. impatiens* could not be obtained across many hours of sampling were sampled for 5 or 10 bees. If a site yielded < 5 *B. impatiens* workers across several hours of sampling, it was not included in the study, thus some of the poorest quality sites were potentially excluded. Bees were gathered into vials in groups of five and stored immediately onto dry ice in the field, followed by transfer to a – 80 °C freezer. Most sites thus contained three replicate vials with five individuals each for subsequent pooled pathogen screens.

### Molecular quantification of pathogens

Abdomens were removed from workers while frozen, placed in a 5 ml tube with 5 metal beads and 2.0 ml of Qiazol (2018) or 2.5 ml Trizol (2019) buffer and homogenized using an Omni Bead ruptor for 35 s on ‘low’. After brief centrifugation to remove particulate matter, 350 μl of homogenate was used for RNA extraction. RNA was extracted using the standard protocol for the Direct-zol RNA Miniprep Kit (Zymo Research, Irvine, California) including incorporated DNA removal steps with DNaseI. RNA samples eluted in water were quantified and quality assessed using a Nanodrop One (Thermo Fisher Scientific, Waltham, Massachusetts). Then, 500 ng (2018) or 400 ng (2019) was taken through cDNA synthesis using standard protocols of the High-Capacity cDNA Reverse Transcription Kit (Thermo Fisher Scientific) (10 µl RNA + 10 µl Mastermix reaction, proportional to recommended quantities), followed by a 1(cDNA):4(water) dilution. Quantitative PCR was performed using this cDNA to assess loads *Vairimorpha bombi* (SSU 16S ribosomal RNA, BOMBICAR primers^73,74^) and loads of single stranded positive-sense RNA viruses BQCV and DWV (primers from Ref.^75^). We also quantified expression of the immune gene, *Defensin*, using primers designed for bumble bees and previously used on *B. impatiens* (Bi’def-278F, Bi’def-397R^76^. Bee housekeeping gene Elongation factor 1α (EF-1α) was used as a control gene for normalization (primers sequences and conditions in 78) of immune genes and pathogen loads against available bee tissue. For qPCR, 1 µl of cDNA product, 5 µl of SYBR green, and 0.3 µl each of 10 µM primer were combined and run on an 7900RT Fast Real-time PCR system machine (40 cycles, 60 °C annealing temperature; Applied Biosystems, Waltham, Massachusetts) using dissociation curves to ensure no non-specific products were generated. qPCRs were run in triplicate using standard curves and no template controls. Standard curves included eight serial fivefold dilutions of original PCR amplified or concentrated cDNA product. Within-site samples were randomized across extraction and cDNA runs and plates, with 2018 and 2019 samples run separately.

Extent of gene expression was calculated from qPCR C_T_ values by first adjusting C_T_ values based on inferred amplification efficiency of standard curves. Standard curves showed good primer efficiency for all genes, with linear increase of C_T_ values closely matching doubling of sampling concentration across values. The resulting adjusted values were normalized against the standard curve adjusted expression of the EF-1α control gene (subtracting EF-1α C_T_ values from C_T_ values). Samples which deviated strongly from the others for EF-1α (most samples were within 2 C_T_ values of one another) were removed from analysis. Samples where no expression was detected, which was found in some pathogen samples, were assigned a final C_T_ value of the sample of lowest detected expression level (highest C_T_) rounded up to the nearest integer which included C_T_ cycles of 35 (BQCV), 37 (*Vairimorpha*), and 39 (DWV). For statistical analyses, we used the normalized values (normalized to the amount of EF-1α in each sample) for DWV, BQCV, *Vairimorpha* and defensin. To quantify body size, we measured the marginal (radial) cells on the right forewing of each worker (to the nearest 0.1 mm) using a dissecting microscope^77^.

### Landscape data metrics

We characterized broad land cover categories around each sampling location from the most recent version (2016) of the National Land Cover Database (NLCD^69^, 30 m resolution). Using the *raster* package in program R version 3.6.1^78,79^, we extracted percent area for forest cover (deciduous + mixed + coniferous), developed cover (‘open space’ through-‘intensely developed’), pasture, row crop, and shrubland, within 2 km radius of the starting location for each study site. We also created a category for all cultivated crops combined (a sum of all crop categories; hereafter, ‘arable’ land cover) and all ‘natural’/semi-natural communities combined (e.g., the sum of: forest, shrubland, wetland, etc.; hereafter, ‘natural’ land cover). Although bumble bees are known to occasionally use habitat at broader spatial scales^5,80^, the majority of foraging activity for *B. impatiens* and other bumble bee species likely occurs within 2 km of colonies^81,82^ suggesting this as a useful scale for analysis.

Although we consider broad land cover classes (e.g., NLCD, described above; Fig. 1), a primary interest of ours was to assess the value of published habitat indices of bee habitat quality. Of particular focus herein were the spring and summer floral quality indices published by Koh et al.^54^, their nesting habitat quality index, and the insecticide loading index published by Douglas et al.^14^. To predict floral and nesting resources at each of our study sites, we used the Integrated Valuation of Environmental Services and Tradeoffs (InVEST v. 3.1.0) crop pollination model^83^. The floral and nesting indices available through the InVEST crop pollination model were developed to explain and predict the relative value of different habitats to pollinator communities from the perspectives of floral resource availability and nest site availability^53,82^. These habitat indices were developed by surveying a panel of experts on wild bee ecology regarding their understanding of relative resource quality among vegetation communities in the NLCD. Expert ranks were then averaged across each land cover category to produce a re-class table which can be used to translate the NLCD into maps of predicted habitat quality for components of bee habitat^53^. As in Kammerer et al.^84^, we translated land use into relative value of nesting and floral resources with reclassification tables from Koh et al.^54^, and, for distance-weighting procedures within the model, assumed a *Bombus* foraging range of 2 km. To generate maps of agricultural insecticide loading (bee lethal doses applied per ha), we obtained year × state × crop reclassification tables presented by Douglas et al.^14^ [Douglas et al., unpublished data] through email correspondence with the corresponding author (M. Douglas). As with our floral and nesting indices, we used the Douglas et al. ^14^ reclassification tables to scale land cover into insecticide toxic load and applied a distance-weighting procedure to more heavily weight insecticide application proximate to our study sites. To calculate nesting, floral, and insecticide indices, we used the 2018–19 Cropland Data Layer (USDA NASS 2018) as our land-use layer, which includes natural habitats from the NLCD and greater resolution of agricultural crop types (Ref.^85^; Fig. 1C,D).

In addition to data on cover type and resource availability indices, we incorporated data related to site-specific weather conditions during each sampling year. Specifically, we quantified cumulative precipitation and growing degree day (GDD; base = 0 °C) maps for our study area using the publicly available PRISM daily weather dataset (Ref.^86^; 4 km resolution). From these maps, we extracted precipitation and GDD values at each sampling location for ‘spring’ (March–May) and ‘summer’ (June–August) of each survey year. We quantified honey bee colony density using the Pennsylvania Department of Agriculture registered apiary database (K. Roccasecca, unpub. data) with each apiary buffered by 5 km^49^, scaled by the number of colonies in each apiary (Fig. 1B).

### Statistical analyses

Prior to analysis we scaled all variables around a mean of zero to improve model convergence using the *scale* function in R. We assessed the relationships between bumble bee metrics of pathogen prevalence (*Vairimorpha*, BQCV, DWV, and a combined pathogen metric), expression of the immune gene *Defensin*, and body size, against landscape features described above using an information-theoretic approach^87^. Our use of the information-theoretic approach involved ranking a series of models with the same response variable (hereafter, a ‘set’ of models), each of which is a ‘candidate’ for the position of top rank in a statistical hierarchy, as determined by an information criterion like Akaike’s Information Criterion (AIC^87^). All models presented here are analyzed as ranked candidate model sets. Our combined pathogen metric was the mean normalized pathogen value averaged across our three pathogens, *Vairimorpha*, BQCV, and DWV. Specifically, we developed candidate sets of mixed-effects linear models using the *lme4* package in R^88^. All models included a random effect for ‘site’ to account for potential non-independence of individual samples within each site^89^ and each year was modeled separately. In each model, bumble bee pathogen metrics for each group of five workers were used as response variables with site-specific habitat characteristics as predictors. For *Defensin* expression, we also considered pathogen loads as predictor variables. Within each model set, we considered all possible combinations of 0–2 predictor variables with both linear (x) and quadradic (x^2^) relationships. To assess the extent to which both independent and dependent variables were correlated prior to analyses, we created correlation matrices of all pairwise variables. We considered two variables to be correlated if |r|> 0.70^90^. Because there were numerous covariates that were correlated (Fig. 2) and correlated variables impact numerical optimization of linear models^89^, we specified only models where correlated predictors did not occur together in a single model. Additionally, in all model sets, we also specified a ‘null’ model that included only the random effect for ‘site’ with no fixed effects.

We specified a total of 22 candidate model sets with 11 sets in our first tier and 11 analogous sets in our second tier. The 11 model sets in each tier consisted of our six response variables: (1) *Vairimorpha*, (2) BQCV, (3) DWV, (4) *Defensin*, (5) combined pathogen load, and (6) marginal cell size analyzed separately for each year (marginal cell size only for 2019). Within each candidate model set, we assessed models and associated covariates using AIC adjusted for small sample size (AIC_c_; models < 2.0 AIC_c_ considered equivalent) applying *β* 95% confidence intervals (with those overlapping zero considered weak effects^87^.

## Supplementary information

Supplementary Information.

## Data Availability

Upon acceptance, the datasets generated and analyzed during the current study will be publicly available in the Dryad data repository.

## References

[1] Potts SG (2016). Safeguarding pollinators and their values to human well-being. Nature.

[2] Potts SG (2010). Global pollinator declines: Trends, impacts and drivers. Trends Ecol. Evol..

[3] Cameron SA, Sadd BM (2020). Global trends in bumble bee health. Annu. Rev. Entomol..

[4] Goulson D, Nicholls E, Botías C, Rotheray EL (2015). Bee declines driven by combined stress from parasites, pesticides, and lack of flowers. Science.

[5] Steffan-Dewenter I, Münzenberg U, Bürger C, Thies C, Tscharntke T (2002). Scale-dependent effects of landscape context on three pollinator guilds. Ecology.

[6] Winfree R, Aguilar R, Vázquez DP, LeBuhn G, Aizen MA (2009). A meta-analysis of bees' responses to anthropogenic disturbance. Ecology.

[7] Grozinger CM, Flenniken ML (2019). Bee viruses: Ecology, pathogenicity, and impacts. Annu. Rev. Entomol..

[8] Cameron SA (2011). Patterns of widespread decline in North American bumble bees. Proc. R. Soc. B Biol. Sci..

[9] Tokarev YS (2020). A formal redefinition of the genera Nosema and Vairimorpha (Microsporidia: Nosematidae) and reassignment of species based on molecular phylogenetics. J. Invertebr. Pathol..

[10] Levitt AL (2013). Cross-species transmission of honey bee viruses in associated arthropods. Virus Res..

[11] Radzevičiūtė R (2017). Replication of honey bee-associated RNA viruses across multiple bee species in apple orchards of Georgia, Germany and Kyrgyzstan. J. Invertebr. Pathol..

[12] Fürst MA, McMahon DP, Osborne JL, Paxton RJ, Brown MJF (2014). Disease associations between honeybees and bumblebees as a threat to wild pollinators. Nature.

[13] Dolezal AG (2016). Honey bee viruses in wild bees: Viral prevalence, loads, and experimental inoculation. PLoS ONE.

[14] Douglas MR, Sponsler DB, Lonsdorf EV, Grozinger CM (2020). County-level analysis reveals a rapidly shifting landscape of insecticide hazard to honey bees (*Apis mellifera*) on US farmland. Sci. Rep..

[15] Blacquiere T, Smagghe G, Van Gestel CA, Mommaerts V (2012). Neonicotinoids in bees: A review on concentrations, side-effects and risk assessment. Ecotoxicology.

[16] Aliouane Y (2009). Subchronic exposure of honeybees to sublethal doses of pesticides: effects on behavior. Environ. Toxicol. Chem..

[17] Whitehorn PR, O’connor S, Wackers FL, Goulson D (2012). Neonicotinoid pesticide reduces bumble bee colony growth and queen production. Science.

[18] Soroye P, Newbold T, Kerr J (2020). Climate change contributes to widespread declines among bumble bees across continents. Science.

[19] Dolezal AG, Toth AL (2018). Feedbacks between nutrition and disease in honey bee health. Curr. Opin. Insect Sci..

[20] DeGrandi-Hoffman G, Chen Y (2015). Nutrition, immunity and viral infections in honey bees. Curr. Opin. Insect Sci..

[21] DeGrandi-Hoffman G, Chen Y, Huang E, Huang MH (2010). The effect of diet on protein concentrcation, hypopharyngeal gland development and virus load in worker honey bees (*Apis mellifera* L.). J. Insect Physiol..

[22] Di Pasquale G (2013). Influence of pollen nutrition on honey bee health: Do pollen quality and diversity matter?. PLoS ONE.

[23] Manley R, Boots M, Wilfert L (2017). Condition-dependent virulence of slow bee paralysis virus in *Bombus terrestris*: Are the impacts of honeybee viruses in wild pollinators underestimated?. Oecologia.

[24] Ricigliano VA (2019). Honey bee colony performance and health are enhanced by apiary proximity to US Conservation Reserve Program (CRP) lands. Sci. Rep..

[25] O’Neal ST, Anderson TD, Wu-Smart JY (2018). Interactions between pesticides and pathogen susceptibility in honey bees. Curr. Opin. Insect Sci..

[26] Di Prisco GV (2013). Neonicotinoid clothianidin adversely affects insect immunity and promotes replication of a viral pathogen in honey bees. Proc. Natl. Acad. Sci..

[27] O’Neal ST, Swale DR, Anderson TD (2017). ATP-sensitive inwardly rectifying potassium channel regulation of viral infections in honey bees. Sci. Rep..

[28] Fine JD, Cox-Foster DL, Mullin CA (2017). An inert pesticide adjuvant synergizes viral pathogenicity and mortality in honey bee larvae. Sci. Rep..

[29] Pettis JS, Johnson J, Dively G (2012). Pesticide exposure in honey bees results in increased levels of the gut pathogen *Nosema*. Naturwissenschaften.

[30] Pettis JS, Lichtenberg EM, Andree M, Stitzinger J, Rose R (2013). Crop pollination exposes honey bees to pesticides which alters their susceptibility to the gut pathogen *Nosema ceranae*. PLoS ONE.

[31] McArt SH, Fersch AA, Milano NJ, Truitt LL, Böröczky K (2017). High pesticide risk to honey bees despite low focal crop pollen collection during pollination of a mass blooming crop. Sci. Rep..

[32] McArt SH, Koch H, Irwin RE, Adler LS (2014). Arranging the bouquet of disease: Floral traits and the transmission of plant and animal pathogens. Ecol. Lett..

[33] Piot N (2019). Establishment of wildflower fields in poor quality landscapes enhances micro-parasite prevalence in wild bumble bees. Oecologia.

[34] Bailes EJ (2020). Host density drives viral, but not trypanosome, transmission in a key pollinator. Proc. R. Soc. B Biol. Sci..

[35] Singh R (2010). RNA viruses in hymenopteran pollinators: Evidence of inter-taxa virus transmission via pollen and potential impact on non-*Apis* hymenopteran species. PLoS ONE.

[36] Manley R, Boots M, Wilfert L (2015). Emerging viral disease risk to pollinating insects: Ecological, evolutionary and anthropogenic factors. J. Appl. Ecol..

[37] Meeus I, Pisman M, Smagghe G, Piot N (2018). Interaction effects of different drivers of wild bee decline and their influence on host–pathogen dynamics. Curr. Opin. Insect Sci..

[38] Huang Z (2012). Pollen nutrition affects honey bee stress resistance. Terr. Arthropod. Rev..

[39] Smart M, Pettis J, Rice N, Browning Z, Spivak M (2016). Linking measures of colony and individual honey bee health to survival among apiaries exposed to varying agricultural land use. PLoS ONE.

[40] Danihlík J, Aronstein K, Petřivalský M (2015). Antimicrobial peptides: a key component of honey bee innate immunity: Physiology, biochemistry, and chemical ecology. J. Apic. Res..

[41] Meeus I, Brown MJ, De Graaf DC, Smagghe GUY (2011). Effects of invasive parasites on bumble bee declines. Conserv. Biol..

[42] Vaudo AD, Tooker JF, Grozinger CM, Patch HM (2015). Bee nutrition and floral resource restoration. Curr. Opin. Insect Sci..

[43] Sánchez-Bayo F (2016). Are bee diseases linked to pesticides?—A brief review. Environ. Int..

[44] Beck MA, Levander OA (2000). Host nutritional status and its effect on a viral pathogen. J. Infect. Dis..

[45] Hing S, Narayan EJ, Thompson RA, Godfrey SS (2016). The relationship between physiological stress and wildlife disease: Consequences for health and conservation. Wildl. Res..

[46] Graystock P, Goulson D, Hughes WO (2015). Parasites in bloom: Flowers aid dispersal and transmission of pollinator parasites within and between bee species. Proc. R. Soc. B Biol. Sci..

[47] Sponsler DB, Shump D, Richardson RT, Grozinger CM (2020). Characterizing the floral resources of a North American metropolis using a honey bee foraging assay. Ecosphere.

[48] Williams NM, Regetz J, Kremen C (2012). Landscape-scale resources promote colony growth but not reproductive performance of bumble bees. Ecology.

[49] Steffan-Dewenter I, Tscharntke T (2000). Resource overlap and possible competition between honey bees and wild bees in central Europe. Oecologia.

[50] Tehel A, Brown MJ, Paxton RJ (2016). Impact of managed honey bee viruses on wild bees. Curr. Opin. Virol..

[51] Sponsler DB (2019). Pesticides and pollinators: A socioecological synthesis. Sci. Total Environ..

[52] McCaskill GL (2009). Pennsylvania’s Forests 2009.

[53] Park MG, Blitzer EJ, Gibbs J, Losey JE, Danforth BN (2015). Negative effects of pesticides on wild bee communities can be buffered by landscape context. Proc. R. Soc. B Biol. Sci..

[54] Koh I (2016). Modeling the status, trends, and impacts of wild bee abundance in the United States. Proc. R. Soc. B Biol. Sci..

[55] Williams PH, Thorp RW, Richardson LL, Colla SR (2014). Bumble Bees of North America: An Identification Guide.

[56] National Research Council (2001). Under the Weather: Climate, Ecosystems, and Infectious Disease.

[57] Polgreen PM, Polgreen EL (2018). Infectious diseases, weather, and climate. Clin. Infect. Dis..

[58] Retschnig G, Williams GR, Schneeberger A, Neumann P (2017). Cold ambient temperature promotes *Nosema* spp. intensity in honey bees (*Apis mellifera*). Insects.

[59] Dalmon A, Peruzzi ML, Conte Y, Alaux C, Pioz M (2019). Temperature-driven changes in viral loads in the honey bee *Apis mellifera*. J. Invertebr. Pathol..

[60] Gardner WA, Sutton RM, Noblet R (1977). Persistence of *Beauveria bassiana, Nomuraea rileyi*, and *Nosema necatrix* on Soyhean Foliage. Environ. Entomol..

[61] Neidel V, Steyer CS, C., & Hoch, G. (2017). Simulation of rain enhances horizontal transmission of the microsporidium *Nosema lymantriae* via infective feces. J. Invertebr. Pathol..

[62] Rangel J (2017). Prevalence of Nosema species in a feral honey bee population: A 20-year survey. Apidologie.

[63] Leather SR (2017). “Ecological Armageddon”-more evidence for the drastic decline in insect numbers. Ann. Appl. Biol..

[64] Scheper J (2015). Local and landscape-level floral resources explain effects of wildflower strips on wild bees across four European countries. J. Appl. Ecol..

[65] Rodríguez JP, Brotons L, Bustamante J, Seoane J (2017). The application of predictive modelling of species distribution to biodiversity conservation. Divers. Distrib..

[66] Young BE (2019). Using citizen science data to support conservation in environmental regulatory contexts. Biol. Conserv..

[67] Lesley JP (1892). A Summary Description of the Geology of Pennsylvania.

[68] Dyer J (2006). Revisiting the Deciduous Forests of Eastern North America. Bioscience.

[69] Wherry, E. T., Fogg, Jr., J. M., & Wahl. H. A. *Atlas of the Flora of Pennsylvania*. (University of Pennsylvania, Pennsylvania, 1979).

[70] Albright, T. A. *Forests of Pennsylvania, 2017*. Resource Update FS-175. (U.S. Department of Agriculture, Forest Service, 2017).

[71] Wickham J (2014). The multi-resolution land characteristics (MRLC) consortium—20 years of development and integration. Remote Sens..

[72] Shannon CE (1948). A mathematical theory of communication. Bell Labs Tech. J..

[73] Plischuk S (2009). South American native bumblebees (Hymenoptera: Apidae) infected by *Nosema ceranae* (Microsporidia), an emerging pathogen of honeybees (*Apis mellifera*). Environ. Microbiol. Rep..

[74] Chu CC, Cameron SA (2017). A scientific note on *Nosema bombi* infection intensity among different castes within a *Bombus auricomus* nest. Apidologie.

[75] vanEngelsdorp D (2009). Colony collapse disorder: A descriptive study. PLoS ONE.

[76] Simmons WR, Angelini DR (2017). Chronic exposure to a neonicotinoid increases expression of antimicrobial peptide genes in the bumblebee *Bombus impatiens*. Sci. Rep..

[77] Muller CB, Schmid-Hempel P (1992). Variation in life-history pattern in relation to worker mortality in the bumble-bee, *Bombus lucorum*. Funct. Ecol..

[78] Hijmans, R. J. & van Etten, J. Raster: Geographic analysis and modeling with raster data. *R package version 2.0-12.* http://CRAN.R-project.org/package=raster (2012).

[79] R Core Team. R: A language and environment for statistical computing. R Foundation for Statistical Computing, Vienna, Austria. http://www.r-project.org/index.html (2019).

[80] Knight ME (2009). Bumblebee nest density and the scale of available forage in arable landscapes. Insect Conserv. Diver..

[81] Darvill B, Knight ME, Goulson D (2004). Use of genetic markers to quantify bumblebee foraging range and nest density. Oikos.

[82] Desjardins ÈC, De Oliveira D (2006). Commercial bumble bee *Bombus impatiens* (Hymenoptera: Apidae) as a pollinator in lowbush blueberry (Ericale: Ericaceae) fields. J. Econ. Entomol..

[83] Natural Capital Project. InVEST: Crop Pollination Model. Version 3.1.0. http://naturalcapitalproject.org/models/crop_pollination.html (2014).

[84] Kammerer MA, Biddinger DJ, Joshi NK, Rajotte EG, Mortensen DA (2016). Modeling local spatial patterns of wild bee diversity in Pennsylvania apple orchards. Landsc. Ecol..

[85] Johnson DM, Mueller R (2010). The 2009 cropland data layer. Photogramm. Eng. Remote. Sens..

[86] PRISM Climate Group. PRISM Gridded Climate Data. Oregon State University, Corvallis Oregon, USA. http://prism.oregonstate.edu (2019).

[87] Burnham KP, Anderson DR (2002). Model Selection and Multimodel Inference—A Practical Information-Theoretic Approach.

[88] Bates D, Mächler M, Bolker BM, Walker S (2015). Fitting linear mixed-effects models using *lme4*. J. Stat. Softw..

[89] Zuur A, Ieno EN, Walker N, Saveliev AA, Smith GM (2009). Mixed Effects Models and Extensions in Ecology with R.

[90] Sokal RR, Rohlf FJ (1969). The Principles and Practice of Statistics in Biological Research.

