# Supplementary material for: Bumble bees in landscapes with abundant floral resources have lower pathogen loads
Source: Sci Rep. 2020 Dec 18;10:22306. doi: 10.1038/s41598-020-78119-2 (PMC7749142; doi:10.1038/s41598-020-78119-2)
Supplement: Supplementary file 1 — Supplementary Information. [file 41598_2020_78119_MOESM1_ESM.docx]

**SUPPLEMENTARY INFORMATION**

**Title:**

Bumble Bees in Landscapes with Abundant Floral Resources Have Lower Pathogen Loads

**Authors:**

Darin J. McNeil, Elyse McCormick, Ashley Heimann, Melanie Kammerer, Margaret Douglas, Sarah C. Goslee, Christina M. Grozinger, Heather M. Hines

**Supplementary Table S1.** Mixed-effects models of *Defensin* expression corrected for the EF-1α housekeeping gene in *Bombus impatiens* workers collected across varied landscapes in Pennsylvania 2018-19. Each year was modeled separately and models were specified in two model tiers: Tier 1 included covariates related to our primary objectives while Tier 2 assessed secondary covariates of interest. For each model we include the number of model parameters (k), Δ Akaike’s Information Criterion adjusted for small sample size (ΔAIC_c_) and model weight (*w*). All model sets also included a null model with no covariates for comparison. A full list of covariates can be found in Methods. Only shown are the competing models (ΔAICc < 2.0 from the top model). For each covariate, *β* coefficient signs are represented as “(+)” for positive, “(-)” for negative, and depicted with no symbol when *β* 95% confidence intervals included zero indicating weak biological effects.

| ***Defensin* expression 2018 – Tier 1** | | | |
| --- | --- | --- | --- |
| Model | k | ΔAIC_c_ | *w* |
| *Null* | 3 | 0.00 | 0.07 |
| Insecticide loading | 4 | 0.04 | 0.07 |
| Insecticide loading^2^ | 5 | 0.53 | 0.05 |
| Spring floral resources | 4 | 0.55 | 0.05 |
| Honey bee colony density | 4 | 0.67 | 0.05 |
| Insecticide loading + Spring floral resources | 5 | 1.34 | 0.04 |
| Insecticide loading + Honey bee colony density | 5 | 1.37 | 0.03 |
| Nesting resources | 4 | 1.55 | 0.03 |
| Insecticide loading + Summer floral resources | 5 | 1.77 | 0.03 |
| Insecticides loading^2^ + Honey bee colony density | 6 | 1.77 | 0.03 |
| Honey bee colony density + Spring floral resources | 5 | 1.77 | 0.03 |
| Insecticide loading + Nesting resources | 5 | 1.81 | 0.03 |
| Summer floral resources | 4 | 1.86 | 0.03 |
| Insecticide loading^2^ + Summer floral resources^2^ | 7 | 1.88 | 0.03 |
| Insecticide loading ^2^ + Spring floral resources | 6 | 1.96 | 0.03 |
| ***Defensin* expression 2018 – Tier 2** | | | |
| Grassland/pasture cover + *Nosema* load | 5 | 0.00 | 0.02 |
| Grassland/pasture cover(+) | 4 | 0.59 | 0.01 |
| Grassland/pasture cover(+) + BQCV load | 6 | 1.25 | 0.01 |
| *Nosema* load(+) | 4 | 1.50 | 0.01 |
| Grassland/pasture cover^2^ + Spring GDD^2^ | 7 | 1.71 | 0.01 |
| Arable cover + *Nosema* load | 5 | 1.78 | 0.01 |
| Grassland/pasture cover (+) + Spring GDD^2^ | 6 | 1.94 | 0.01 |
| Latitude + Grassland/pasture cover(+) | 5 | 1.96 | 0.01 |
| Shrubland cover + Grassland/pasture cover (+) | 5 | 1.98 | 0.01 |
|  |  |  |  |
| ***Defensin* expression 2019 – Tier 1** | | | |
| *Null* | 3 | 0.00 | 0.37 |
| ***Defensin* expression 2019 – Tier 2** | | | |
| *Null* | 3 | 0.00 | 0.04 |
| Spring Precipitation | 4 | 1.45 | 0.02 |
| *Nosema* load | 4 | 1.68 | 0.02 |
| Spring Precipitation + Elevation | 5 | 1.70 | 0.02 |

**Supplementary Table S2.** Mixed-effects models of black queen cell virus loads in *Bombus impatiens* workers (corrected for the ef1α housekeeping gene) collected across varied landscapes in Pennsylvania 2018-19. Each year was modeled separately and models were specified in two model tiers: Tier 1 included covariates related to our primary objectives while Tier 2 assessed secondary covariates of interest. For each model we include the number of model parameters (k), Δ Akaike’s Information Criterion adjusted for small sample size (ΔAIC_c_) and model weight (*w*). All model sets also included a null model with no covariates for comparison. A full list of covariates can be found in Methods. Only shown are the competing models (ΔAICc < 2.0 from the top model). For each covariate, *β* coefficient signs are represented as “(+)” for positive, “(-)” for negative, and depicted with no symbol when *β* 95% confidence intervals included zero indicating weak biological effects.

| **Black Queen Cell Virus 2018 – Tier 1** | | | |
| --- | --- | --- | --- |
| Model | k | ΔAIC_c_ | *w* |
| Nesting resources(-) | 4 | 0.00 | 0.17 |
| Spring floral resources(-) | 4 | 0.13 | 0.16 |
| Honey bee colony density + Nesting resources(-) | 5 | 0.80 | 0.11 |
| Nesting resources^2^(-) | 5 | 1.32 | 0.09 |
| **Black Queen Cell Virus 2018 – Tier 2** | | | |
| Latitude^2^+ Forest cover(-) | 6 | 0.00 | 0.07 |
| Longitude(+) + Forest cover(-) | 5 | 0.44 | 0.06 |
| Latitude(-) + Natural cover(-) | 5 | 0.91 | 0.04 |
| Latitude(-) + Developed cover^2^(+) | 6 | 1.05 | 0.04 |
| Latitude^2^ + Natural cover(-) | 6 | 1.10 | 0.04 |
| Forest cover(-) | 4 | 1.11 | 0.04 |
| Spring floral resources(-) + Developed cover^2^(+) | 6 | 1.42 | 0.03 |
| Latitude(-) + Forest cover(-) | 5 | 1.51 | 0.03 |
| Spring floral resources(-) + Developed cover^2^(+) | 6 | 1.76 | 0.03 |
| Longitude(+) + Spring floral resources(-) | 5 | 1.81 | 0.03 |
|  |  |  |  |
| **Black Queen Cell Virus 2019 – Tier 1** | | | |
| Honey bee colony density(+) + Spring floral resources(-) | 5 | 0 | 0.4 |
| **Black Queen Cell Virus 2019 – Tier 2** | | | |
| Spring GDD^2^(+) + Honey bee colony density(+) | 6 | 0.00 | 0.17 |
| Spring floral resources(-) + Honey bee colony density(+) | 5 | 0.46 | 0.13 |
| Shrubland cover (-) + Honey bee colony density(+) | 5 | 1.31 | 0.09 |

**Supplementary Table S3.** Mixed-effects models of *Vairimorpha* infection loads in *Bombus impatiens* workers (corrected for the EF-1α housekeeping gene) collected across varied landscapes in Pennsylvania 2018-19. Each year was modeled separately and models were specified in two model tiers: Tier 1 included covariates related to our primary objectives while Tier 2 assessed secondary covariates of interest. For each model we include the number of model parameters (k), Δ Akaike’s Information Criterion adjusted for small sample size (ΔAIC_c_) and model weight (*w*). All model sets also included a null model with no covariates for comparison. A full list of covariates can be found in Methods. Only shown are the competing models (ΔAICc < 2.0 from the top model). For each covariate, *β* coefficient signs are represented as “(+)” for positive, “(-)” for negative, and depicted with no symbol when *β* 95% confidence intervals included zero indicating weak biological effects.

| ***Vairimorpha* infection 2018 – Tier 1** | | | |
| --- | --- | --- | --- |
| Model | k | ΔAIC_c_ | *w* |
| *Null* | 3 | 0.00 | 0.21 |
| Insecticide loading | 4 | 0.40 | 0.17 |
| Honey bee colony density | 4 | 0.99 | 0.13 |
| ***Vairimorpha* infection 2018 – Tier 2** | | | |
| Arable cover**(+)** | 4 | 0.00 | 0.05 |
| *Null* | 3 | 0.83 | 0.03 |
| *Bombus* spp. Diversity **(-)** | 4 | 1.09 | 0.03 |
| Insecticide loading | 4 | 1.23 | 0.03 |
| Arable**(+)** + *Bombus* spp. diversity | 5 | 1.31 | 0.03 |
| Grassland/pasture2**(+)** + *Bombus* spp. diversity **(-)** | 6 | 1.79 | 0.02 |
| Honey bee colony density | 4 | 1.82 | 0.02 |
| *Bombus* spp. diversity**(-)** + Honey bee colony density **(+)** | 5 | 1.97 | 0.02 |
|  |  |  |  |
| ***Vairimorpha* infection 2019 – Tier 1** | | | |
| *Null* | 3 | 0.00 | 0.50 |
| ***Vairimorpha* infection 2019 – Tier 2** | | | |
| Spring precipitation**(+)** | 4 | 0.00 | 0.14 |

**Supplementary Table S4.** Mixed-effects models of deformed wing virus loads in *Bombus impatiens* workers (corrected for the EF-1α housekeeping gene) collected across varied landscapes in Pennsylvania 2018-19. Each year was modeled separately and models were specified in two model tiers: Tier 1 included covariates related to our primary objectives while Tier 2 assessed secondary covariates of interest. For each model we include the number of model parameters (k), Δ Akaike’s Information Criterion adjusted for small sample size (ΔAIC_c_) and model weight (*w*). All model sets also included a null model with no covariates for comparison. A full list of covariates can be found in Methods. Only shown are the competing models (ΔAICc < 2.0 from the top model). For each covariate, *β* coefficient signs are represented as “(+)” for positive, “(-)” for negative, and depicted with no symbol when *β* 95% confidence intervals included zero indicating weak biological effects.

| **Deformed Wing Virus 2018 – Tier 1** | | | |
| --- | --- | --- | --- |
| Model | k | ΔAIC_c_ | *w* |
| *Null* | 3 | 0.00 | 0.31 |
| **Deformed Wing Virus 2018– Tier 2** | | | |
| Longitude(+) + Summer floral resources(-) | 5 | 0.00 | 0.15 |
| Longitude(+) | 4 | 0.98 | 0.09 |
|  |  |  |  |
| **Deformed Wing Virus 2019 – Tier 1** | | | |
| Insecticide loading^2^(**-**) + Nesting resources(-) | 6 | 0.00 | 0.29 |
| Nesting resources(**-**) | 4 | 0.33 | 0.24 |
| Insecticide loading + Nesting resources(-) | 5 | 1.91 | 0.11 |
| **Deformed Wing Virus 2019– Tier 2** | | | |
| Latitude(**-**) + Arable cover(**-**) | 5 | 0.00 | 0.13 |
| Latitude(**-**) + Insecticide loading^2^(**-**) | 6 | 1.23 | 0.07 |
| Latitude(**-**) | 4 | 1.58 | 0.06 |
| Latitude(**-**) + Arable cover^2^(**-**) | 6 | 1.72 | 0.06 |
| Latitude(**-**) + Natural cover^2^ | 6 | 1.98 | 0.05 |

**Supplementary Table S5.** Mixed-effects models of our combined pathogen index in *Bombus impatiens* workers collected across varied landscapes in Pennsylvania 2018-19. Each year was modeled separately and models were specified in two model tiers: Tier 1 included covariates related to our primary objectives while Tier 2 assessed secondary covariates of interest. For each model we include the number of model parameters (k), Δ Akaike’s Information Criterion adjusted for small sample size (ΔAIC_c_) and model weight (*w*). All model sets also included a null model with no covariates for comparison. A full list of covariates can be found in Methods. Only shown are the competing models (ΔAICc < 2.0 from the top model). For each covariate, *β* coefficient signs are represented as “(+)” for positive, “(-)” for negative, and depicted with no symbol when *β* 95% confidence intervals included zero indicating weak biological effects.

| **Combined pathogen index 2018 – Tier 1** | | | |
| --- | --- | --- | --- |
| Model | k | ΔAIC_c_ | *w* |
| Honey bee colony density(+) + Spring floral resources | 5 | 0.00 | 0.13 |
| Honey bee colony density(+) + Nesting resources | 5 | 0.21 | 0.11 |
| Spring floral resources(-) | 4 | 0.63 | 0.09 |
| Honey bee colony density(+) | 4 | 0.83 | 0.08 |
| **Combined pathogen index 2018 – Tier 2** | | | |
| Longitude(+) + Spring floral(-) | 5 | 0.00 | 0.31 |
|  |  |  |  |
| **Combined pathogen index 2019 – Tier 1** | | | |
| Honey bee colony density + Nesting resources (-) | 5 | 0.00 | 0.18 |
| Nesting resources(-) | 4 | 0.41 | 0.15 |
| Summer Floral + Nesting resources(-) | 5 | 1.84 | 0.07 |
| **Combined pathogen index 2019 – Tier 2** | | | |
| Latitude(-) + Honey bee colony density | 5 | 0.00 | 0.06 |
| Latitude(-) | 4 | 0.27 | 0.05 |
| Latitude(-) + Forest cover | 5 | 0.80 | 0.04 |
| Latitude(-) + Longitude^2^ | 6 | 1.01 | 0.04 |
| Latitude(-) + Nesting resources | 5 | 1.10 | 0.03 |
| Latitude(-) + Spring Precipitation^2^ | 6 | 1.32 | 0.03 |
| Latitude(-) + Honey bee colony density^2^ | 6 | 1.50 | 0.03 |
| Latitude(-) + Spring Precipitation | 5 | 1.70 | 0.03 |

**Supplementary Table S6.** Mixed-effects models of forewing marginal cell length in *Bombus impatiens* workers collected across varied landscapes in Pennsylvania in 2019. Models were specified in two model tiers: Tier 1 included covariates related to our primary objectives while Tier 2 assessed secondary covariates of interest. For each model we include the number of model parameters (k), Δ Akaike’s Information Criterion adjusted for small sample size (ΔAIC_c_) and model weight (*w*). All model sets also included a null model with no covariates for comparison. A full list of covariates can be found in Methods. Only shown are the competing models (ΔAICc < 2.0 from the top model). For each covariate, *β* coefficient signs are represented as “(+)” for positive, “(-)” for negative, and depicted with no symbol when *β* 95% confidence intervals included zero indicating weak biological effects.

| **Marginal cell length 2019 – Tier 1** | | | |
| --- | --- | --- | --- |
| Model | k | ΔAIC_c_ | *w* |
| *Null* | 3 | 0.00 | 0.19 |
| Spring floral resources | 4 | 1.44 | 0.09 |
| Nesting resources | 4 | 1.77 | 0.08 |
| **Marginal cell length 2019 – Tier 2** | | | |
| *Null* | 3 | 0.00 | 0.02 |
| *Bombus* spp. diversity | 4 | 0.70 | 0.01 |
| Spring floral resources | 4 | 1.44 | 0.01 |
| BQCV load | 4 | 1.57 | 0.01 |
| Insecticide loading^2^ + *Bombus* spp. diversity | 6 | 1.76 | 0.01 |
| Nesting resources | 4 | 1.77 | 0.01 |
| Natural cover | 4 | 1.82 | 0.01 |
